# Occult cerebral hemorrhage induced by high-dose oral methanol poisoning: a case report

**DOI:** 10.3389/fphar.2026.1757281

**Published:** 2026-01-26

**Authors:** Yuncheng Tang, Pengfei Liang, Qiming Fang, Juan Zhao, Li Zhang, Xiaolin Zhang, Haowen Tang, Yu Jiang, Zhiwen Zhao, Bo Wang, Zhicheng Fang

**Affiliations:** 1 Department of Emergency Medicine, Taihe Hospital, Hubei University of Medicine, Shiyan, Hubei, China; 2 Department of Radiology, Taihe Hospital, Hubei University of Medicine, Shiyan, Hubei, China; 3 Hubei Provincial Clinical Research Center for Pneumoconiosis and Poisoning, Hubei Provincial Hospital of Integrated Chinese & Western Medicine, Wuhan, Hubei, China; 4 Department of Pediatric Surgery, Taihe Hospital, Hubei University of Medicine, Shiyan, Hubei, China

**Keywords:** basal ganglia, hemodialysis, intracerebral hemorrhage, methanol poisoning, ventricular drainage

## Abstract

**Background:**

Acute methanol intoxication is life-threatening and typically presents with severe metabolic acidosis and central nervous system injury. Intracranial hemorrhage associated with methanol poisoning is uncommon and may be clinically occult, potentially delaying diagnosis and intervention.

**Case:**

We report a case of high-dose oral methanol poisoning presenting with profound metabolic acidosis and coma. Emergent cranial CT revealed basal ganglia hemorrhage. The patient underwent intermittent hemodialysis and received supportive pharmacotherapy. Owing to obstructive hydrocephalus and neurological deterioration, frontal burr-hole drilling and ventricular drainage were performed. Although metabolic derangements partially improved, the patient remained comatose after 1 week of hospitalization. The family requested transfer to another hospital for further management; however, the patient died during inter-hospital transport.

**Conclusion:**

Cerebral hemorrhage secondary to methanol poisoning can be insidious and may occur within hours to days after ingestion. Early neuroimaging and timely intervention are crucial when neurological deterioration or hydrocephalus is suspected.

## 1 Introduction

Methanol (CH_3_OH) is a common industrial solvent widely used in industries such as chemistry, pharmaceuticals, and cosmetics (Cousins, 2025). Due to its similarity in appearance and odor to ethanol, acute poisoning often results from accidental ingestion, consumption of adulterated alcoholic beverages, or intentional intake. The global incidence of methanol poisoning is rising, exhibiting a fluctuating upward trend (Cai et al., 2025; Taylor, 2025) Although methanol itself has relatively low toxicity, its metabolites formaldehyde and formic acid are considerably more toxic, being 20 times and 6 times more toxic than methanol, respectively (Brent et al., 2001). Formaldehyde is metabolized rapidly, whereas formic acid is metabolized more slowly. After ingestion, methanol is first converted to formaldehyde and then further metabolized into formic acid (Brent, 2009). The accumulation of formic acid in the body leads to cellular damage and is the primary toxic agent (Perkins et al., 2025). The minimum lethal dose of methanol is approximately 1 g/kg, and the peak blood concentration typically occurs within 30–90 min after ingestion (Alrashed et al., 2024; Liberski et al., 2022). Due to its small molecular size and lipid solubility, methanol can easily cross the blood-brain barrier (Dorokhov et al., 2015). When the serum methanol concentration exceeds 20 mg/dL (6.24 mmol/L) (Md Noor et al., 2020), classic symptoms of methanol poisoning such as metabolic acidosis, optic nerve injury, and central nervous system depression emerge. Some symptoms may not become evident until 72 h after ingestion (Arslan et al., 2021; Briones-Claudett et al., 2025).

Although standard treatments for methanol poisoning, including hemodialysis, correction of acidosis, and administration of ethanol, have significantly improved patient survival rates, methanol poisoning remains a major clinical challenge. This is largely due to delayed diagnoses, inadequate treatment, and insufficient awareness of potential complications. This article analyzes a case of high-dose oral methanol poisoning complicated by occult cerebral hemorrhage, aiming to highlight the unique aspects of cerebral hemorrhage following methanol poisoning and stress the importance of comprehensive assessment and intervention during the acute phase of poisoning.

## 2 Diagnosis and treatment process

### 2.1 Case description

The patient, a 50-year-old male, was referred to our hospital due to a headache after alcohol consumption for 2 days, accompanied by blurred vision and altered consciousness for the past 14 h. According to the family, the patient consumed approximately 250 mL of white spirit (with unknown alcohol concentration and composition) 2 days ago. After drinking, he developed symptoms including headache, dizziness, a sensation of pressure on the head, along with fatigue and difficulty concentrating. However, no medical attention was sought at that time. Fourteen hours ago, the patient suddenly experienced blurred vision in both eyes, accompanied by photophobia, tearing, headache, nausea, and frothing at the mouth. These symptoms gradually worsened, leading to altered consciousness, confusion, slow responses, rapid breathing, and restlessness. The family took the patient to a local medical facility, where an arterial blood gas analysis was conducted. The results were as follows: pH < 6.8, PaCO_2_ 37 mmHg, PaO_2_ 239 mmHg, Na^+^ 130 mmol/L, K^+^ 5.0 mmol/L, Ca^2+^ 1.04 mmol/L, Glu 24.5 mmol/L, Lac 12.4 mmol/L, Hct 52%, Hb 16.1 g/dL. Blood routine: White blood cell count 19.84 × 10^^9^/L, platelet count 515 × 10^^9^/L, hematocrit 0.54. Renal function: Prealbumin 470.00 mg/L, creatinine 175.0 μmol/L, uric acid 648.0 μmol/L. Myocardial enzyme spectrum: Creatine kinase 97 U/L, creatine kinase isoenzyme 34 U/L, lactate dehydrogenase 256 U/L, α-carboxybutyrate dehydrogenase 175 U/L, myoglobin 292 μg/L, troponin 0.10 ng/mL. The patient received treatments such as tracheal intubation, ventilator-assisted breathing, intravenous high-dose methylprednisolone (200 mg), and blood purification in the emergency room. Due to the prolonged poisoning period, toxins had not been removed within the optimal time frame, and gastrointestinal decontamination (such as gastric lavage, emesis induction, and activated charcoal adsorption of methanol) was not performed. The patient was transferred to the EICU of our hospital for continued treatment 14 h later. The patient has a 2-year history of hypertension, with the highest systolic blood pressure recorded at 180 mmHg.

### 2.2 Physical examination upon admission

Body temperature: 36.6 °C, pulse: 139 beats per minute, respiration: 19 breaths per minute, blood pressure: 189/101 mmHg, SpO2: 99% (tracheal intubation, assisted ventilation with invasive ventilator, P-A/C mode, PS: 18 cmH_2_O, PEEP: 5 cmH_2_O, F: 16 breaths per minute, FiO_2_: 50%). The patient was in a deep coma, with a Glasgow Coma Scale (GCS) score of 4 (E: 1, V: 1, T: 1, M: 1) and an APACHE II score of 32. Physical examination revealed no cooperation. Both pupils were of equal size (approximately 3.5 mm in diameter) with no light reflex. The examination of limb muscle strength was uncooperative, and no localized signs were noted in other physical examinations.

### 2.3 Laboratory tests and imaging evaluation

**FIGURE 1:**
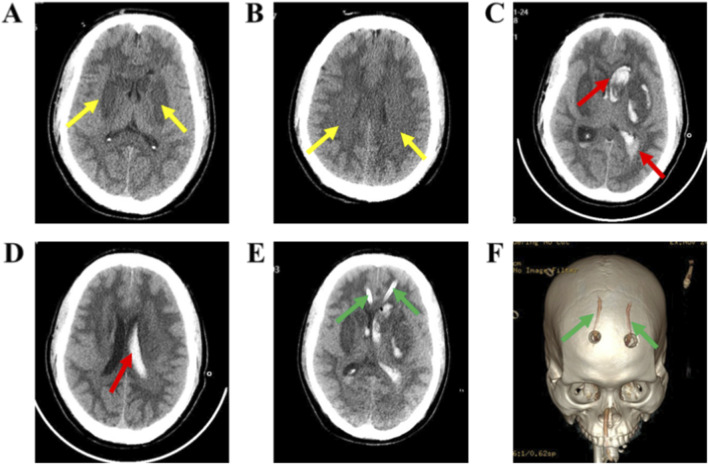
Dynamic changes on cranial CT during treatment. Initial non-contrast CT on admission **(A,B)**: **(A)** Axial non-contrast CT shows symmetric hypoattenuation in the bilateral basal ganglia, predominantly involving the lentiform nuclei (yellow arrows). **(B)** Axial non-contrast CT shows patchy hypoattenuation in the deep white matter of both cerebral hemispheres at the level of the corona radiata/centrum semiovale (yellow arrows). Follow-up CT after clinical deterioration **(C,D)**: **(C)** Axial non-contrast CT demonstrates an acute intracerebral hematoma in the left basal ganglia (predominantly the lentiform nucleus) with intraventricular extension and associated intraventricular hemorrhage; marked mass effect is present, including ventricular compression, midline shift, and effacement of sulci and basal cisterns, indicating a risk of herniation. **(D)** Repeat axial non-contrast CT shows interval evolution of the left basal ganglia hematoma and intraventricular hemorrhage (red arrow), with a corresponding change in mass effect. Postoperative CT **(E,F)**: **(E)** Postoperative axial non-contrast CT shows bilateral frontal external ventricular drains (EVDs; green arrows) with catheter tips positioned in the frontal horns of the lateral ventricles; residual intraventricular hemorrhage persists. **(F)** Three-dimensional CT reconstruction depicts the bilateral frontal burr holes/catheter entry sites (green arrows), corresponding to the surgical drainage tract.

The results of arterial blood gas analysis in our hospital’s emergency room were as follows: pH: 7.35, PaCO_2_: 23.0 mmHg, PaO_2_: 125.2 mmHg, Na^+^: 153.2 mmol/L, K^+^: 3.6 mmol/L, Ca^2+^: 1.2 mmol/L, Glu: 6.9 mmol/L, BE (B): −10.6 mmol/L, SO2: 99.5%, THbc: 15.9 g/dL, Lac: 5.4 mmol/L. Figures 1A,B: Cranial CT scans show patchy low-density shadows in the deep white matter of both cerebral hemispheres. Chest CT revealed inflammatory infiltrates in both lungs, with patchy ground-glass density shadows in the lower lobe of the right lung and blurred margins. No significant organic lesions were found on the full abdominal CT.

### 2.4 Diagnosis and treatment measures

Based on the patient’s recent history of alcohol consumption, accompanied by metabolic acidosis, central nervous system symptoms, and optic nerve damage, along with the blood gas analysis (elevated anion gap), methanol poisoning was diagnosed. The patient presented with significant metabolic acidosis, which likely reduced the body’s ability to clear formic acid. Methanol, formaldehyde, and formic acid are all water-soluble small molecules, and blood purification can aid in removing methanol and its metabolites. Given the patient’s hemodynamic stability, intermittent hemodialysis (IHD) was initially implemented to remove toxins. Pre-purification test results showed a blood methanol concentration of 41.33 mmol/L and an ethanol concentration of less than 0.01 mg/100 mL.

For toxic encephalopathy, a stepwise, targeted treatment strategy was employed. Physical cooling with ice caps was used to reduce brain metabolism and protect neurons. Mannitol (1.0 g/kg, intravenous injection every 12 h) was used to rapidly decrease intracranial pressure and prevent cerebral edema and brain herniation. Edaravone (0.5 mg/kg, intravenous injection every 12 h) was administered as a neuroprotective agent.

Considering folic acid’s role in formic acid metabolism, folic acid (1 mg/kg, intravenous injection every 6 h) was also administered. Additionally, glucocorticoids (methylprednisolone 250 mg, intravenous injection every 6 h) were used to suppress pro-inflammatory factors and protect the nervous system. To protect glial cells and promote anti-inflammatory, anti-apoptotic, and antioxidant effects, recombinant erythropoietin (10,000 IU, subcutaneous injection every 12 h) was administered.

To prevent liver damage caused by toxins, reduced glutathione, vitamin C, and B vitamins were given to enhance the liver’s detoxification and antioxidant capabilities. All treatment measures were aimed at stabilizing the internal environment (with careful fluid replacement) and preventing stress ulcers (using PPIs for acid suppression). For pulmonary infections, antibiotics were administered, and airway management (including airway dilation, nebulization therapy, and bronchoscopy lavage when necessary) was used to ensure airway patency and control infection. Soft gauze was applied to protect the eyes from light exposure, and dynamic monitoring was conducted.

**FIGURE 2:**
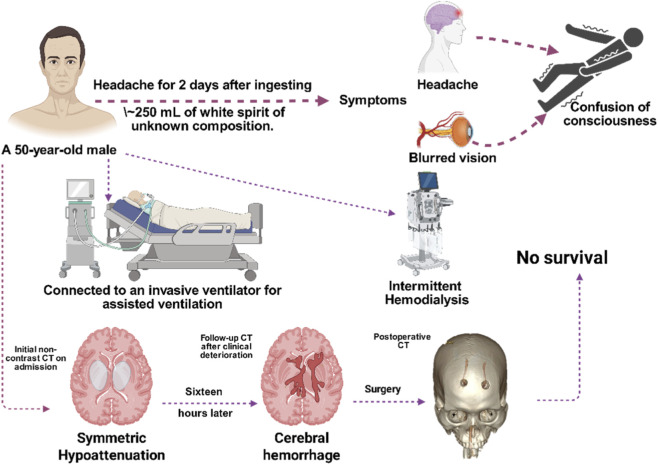
The inpatient treatment and prognosis timeline of the patient.

Sixteen hours later, the patient experienced intermittent limb tremors, accelerated heart rate, irregular breathing patterns, dilated pupils, and loss of the light reflex, suggesting the possible onset of intracranial hemorrhage. As shown in Figures 1C,D, cranial CT revealed that the hematoma in the left basal ganglia and corona radiata had broken into the ventricles, accompanied by brain herniation and multiple low-density areas in the brain. The cerebrovascular team recommended performing intracranial hematoma drainage. Twenty hours later, as shown in Figures 1E,F, the patient underwent a frontal drilling and drainage procedure to reduce intracranial pressure and placed a drainage tube to facilitate hematoma clearance. One week after being hospitalized, the patient remained in a coma. The family decided to transfer the patient for further treatment. Unfortunately, the patient died during the transfer. As shown in Figure 2, this chart illustrates the inpatient treatment and prognosis timeline of the patient.

## 3 Discussion

Methanol poisoning is a relatively common cause of acute poisoning in clinical practice, particularly in cases of accidental ingestion or suicidal intake, which often lead to severe damage to multiple organs. Methanol is metabolized into formaldehyde in the body by alcohol dehydrogenase and aldehyde dehydrogenase, which is then further converted into formic acid. Formic acid accumulates rapidly, resulting in metabolic acidosis, cellular damage, organ failure, and potentially fatal consequences.

The clinical symptoms of methanol poisoning depend on the route of exposure and dosage. Inhalation of methanol vapor can cause inflammation of the eyes, nose, and throat, as well as headache and nausea. Oral ingestion of methanol over a period of 0.5–4 h may cause gastrointestinal symptoms (nausea, vomiting, abdominal pain) and central nervous system depression (drowsiness, sleepiness) (Nekoukar et al., 2021). The average incubation period for acute methanol poisoning is between 12 and 24 h. If ethanol is consumed simultaneously, its affinity for alcohol dehydrogenase is 10–20 times that of methanol (Zakharov et al., 2016), which may prolong the incubation period of methanol. The apparent elimination half-life of methanol is 1–3 h in mild cases of poisoning and approximately 24 h in severe cases (Barceloux et al., 2002). Between 90% and 95% of absorbed methanol is metabolized and excreted through exhaled breath and urine, while 2%–5% is excreted in its original form through the kidneys [9]. When ethanol is used as an antidote (with a serum concentration of 1000 mg/L), it can extend the methanol half-life to 40–50 h. Similarly, when fomepizole is used as the detoxifier, it can extend the methanol half-life by 4–6 times (Tomsia et al., 2022).

Early treatment significantly improves clinical outcomes. Intermittent hemodialysis (IHD) is the preferred external treatment for methanol poisoning, as it not only effectively removes methanol and its toxic metabolites, but also corrects acid-base imbalances, thereby reducing mortality (Şahin et al., 2025; Wang et al., 2023). Clinical guidelines recommend considering hemodialysis when a patient’s serum methanol concentration reaches 50 mg/dL (15.6 mmol/L) or higher, accompanied by severe acidosis or visual impairment. In cases of hemodynamic instability, severe cerebral edema, or when hemodialysis is not possible, continuous renal replacement therapy (CRRT) is suggested as an alternative (Brent, 2009). Fomepizole is considered the first-line antidote for methanol poisoning. By inhibiting alcohol dehydrogenase, it blocks the conversion of methanol to formic acid, thus reducing the accumulation of toxic metabolites at the source (Perkins et al., 2025). If fomepizole is unavailable, ethanol can be administered orally or intravenously, with the same mechanism of action (Mahdavi et al., 2022). However, ethanol as an antidote carries risks such as ethanol toxicity and hypoglycemia, and many poisoned individuals have a history of alcohol abuse, necessitating repeated methanol and ethanol concentration testing.

Previous studies have established clinical markers to predict outcomes in methanol poisoning. Research has shown that patients with acute methanol poisoning have higher systemic immune inflammation index (SII) and total immune inflammation value (PIV), both of which are significantly associated with in-hospital mortality. The SII and PIV values in deceased patients are notably higher than in survivors, and these indices have been identified as independent risk factors for death in multivariate Cox regression analysis (Şahin et al., 2025). Additionally, deceased patients often present with altered consciousness, while survivors typically exhibit ocular symptoms. Deceased patients tend to have lower pH and bicarbonate (HCO3) levels, and higher anion gap and lactate levels, which can be used as markers of poor prognosis in clinical practice (Sarıbaş et al., 2025). Recent studies have shown that chemical resistance sensors are highly sensitive and selective in detecting methanol, especially in the presence of high-concentration ethanol, effectively distinguishing methanol from ethanol (Hassan et al., 2025).

The neuroimaging manifestations of methanol poisoning show relatively characteristic MRI features. Bilateral, symmetric involvement of the basal ganglia is common, most typically affecting the putamen. Lesions may reflect ischemic necrosis and cytotoxic edema. On T2-weighted and FLAIR images, the affected areas often demonstrate hyperintensity. In the acute phase, DWI frequently shows diffusion restriction with corresponding ADC reduction. Some cases exhibit hemorrhagic transformation or hemorrhagic necrosis, for which GRE/SWI is more sensitive. Additional findings may include subcortical and deep white-matter lesions, intraventricular hemorrhage, and, in advanced stages, acute obstructive hydrocephalus (Balodis et al., 2024; Jain et al., 2013). Beyond parenchymal brain injury, methanol poisoning can also involve the optic nerves and visual pathways. Cranio-orbital MRI may reveal ischemic changes of the optic nerves, and optic-nerve diffusion restriction on DWI is considered an objective imaging marker closely associated with visual prognosis. Studies suggest that when optic-nerve diffusion restriction observed in the acute phase subsequently resolves during follow-up, visual recovery is more likely, indicating that DWI is useful not only for detecting optic-nerve involvement but also for prognostic stratification (Durmaz et al., 2024). Phenotypic variability remains substantial: visual function may be relatively preserved despite bilateral basal ganglia necrosis and hemorrhage, whereas some patients develop long-term neuro-ophthalmic sequelae, such as glaucoma-like optic disc cupping and visual impairment, underscoring the need for comprehensive assessment combining MRI with ophthalmologic follow-up (Hassanpoor and Niyousha, 2021; Mojica et al., 2020; Pan et al., 2025).

In emergency and critical care settings, CT remains the preferred intracranial imaging modality for suspected methanol poisoning because of its speed and broad availability. It is particularly useful for promptly identifying intracerebral hemorrhage, intraventricular hemorrhage, mass effect/herniation risk, and actionable postoperative findings such as the position of ventricular drainage catheters. However, CT has limited sensitivity for early ischemic changes, cytotoxic edema, microbleeds, and optic-nerve/visual-pathway involvement, which can lead to underestimation of lesion extent and characterization (Balodis et al., 2024; Geibprasert et al., 2010; Liang et al., 2025). By contrast, MRI enables more precise qualitative assessment using T2/FLAIR, DWI/ADC, and GRE/SWI sequences-for example, distinguishing ischemic injury from hemorrhagic transformation and detecting optic-nerve ischemia with implications for visual prognosis. MRI also supports quantitative evaluation and long-term prognostic research. For instance, basal ganglia volumetry can be correlated with indicators of poisoning severity and may be associated with structural visual biomarkers such as retinal nerve fiber layer thickness on OCT, thereby providing more objective quantitative measures for follow-up of central nervous system and visual sequelae (Balodis et al., 2024; Hlusicka et al., 2020). Accordingly, CT can guide rapid decision-making and dynamic monitoring in the acute stage, while cranial and cranio-orbital MRI should be obtained as early as feasible when life-support conditions permit, to comprehensively assess basal ganglia, white matter, and optic-nerve/visual-pathway involvement and to aid prognostic evaluation (Durmaz Engin et al., 2024; Jain et al., 2013).

We fully recognize the educational value of MRI in case reports of methanol poisoning, particularly for demonstrating optic-nerve ischemia and typical basal ganglia injury. However, our patient was critically ill on admission, in deep coma, dependent on invasive mechanical ventilation, and required continuous bedside monitoring. Transport to the MRI suite therefore posed substantial safety risks. In addition, MRI-compatible ventilatory and monitoring equipment was unavailable at the time, making MRI infeasible while maintaining stable life support. Consequently, we relied primarily on serial cranial CT for emergency assessment and dynamic monitoring. These objective constraints prevented us from providing the representative MRI sequences recommended by the reviewers, which constitutes an important limitation of this study.

## 4 Conclusion

Occult cerebral hemorrhage following methanol poisoning is a severe and often insidious complication. Its clinical presentation can be subtle, leading to delayed diagnosis. Therefore, early identification of cerebral hemorrhage and other complications, particularly through close monitoring of the nervous system, is crucial during the acute phase of poisoning. Strengthening imaging examinations, closely monitoring the patient’s condition, and employing treatment measures such as blood purification can effectively reduce mortality rates and adverse outcomes in patients with methanol poisoning.

## Data Availability

The original contributions presented in the study are included in the article/supplementary material, further inquiries can be directed to the corresponding authors.
